# Case report: Long response to PD-1 blockade after failure of trastuzumab plus chemotherapy in advanced Epstein-Barr virus-associated gastric cancer

**DOI:** 10.3389/fimmu.2022.1003859

**Published:** 2022-10-24

**Authors:** Yan Pan, Linbin Lu, Huan Liu, Di Chen, Ning Han, Ruirong Yao, Xinlin Wang, Xianchun Gao, Jun Yu, Ling Chen, Fenli Zhou, Guangjun Hao, Yuanyuan Lu, Mengbin Li, Guangbin He, Fei Kang, Zengshan Li, Yongqiang Tang, Jinsong Zhang, Lichun Wei, Yongzhan Nie

**Affiliations:** ^1^ State Key Laboratory of Cancer Biology and National Clinical Research Center for Digestive Diseases, Xijing Hospital, The Fourth Military Medical University of People's Liberation Army (PLA), Xi’an, China; ^2^ Department of Pathology, Xijing Hospital, The Fourth Military Medical University of People's Liberation Army (PLA), Xi’an, China; ^3^ Department of Oncology, First Hospital of Yulin City, Yan’an University, Yulin, China; ^4^ Department of Gastrointestinal Surgery, Xijing Hospital, The Fourth Military Medical University of People's Liberation Army (PLA), Xi’an, China; ^5^ Department of Ultrasound Diagnostics, Xijing Hospital, The Fourth Military Medical University of People's Liberation Army (PLA), Xi’an, China; ^6^ Department of Nuclear Medicine, Xijing Hospital, The Fourth Military Medical University of People's Liberation Army (PLA), Xi’an, China; ^7^ Department of Radiology, Xijing Hospital, The Fourth Military Medical University of People's Liberation Army, Xi’an, China; ^8^ Department of Radiotherapy, Xijing Hospital, The Fourth Military Medical University of People's Liberation Army, Xi’an, China

**Keywords:** gastric cancer, HER2, PD-1, Epstein-Barr virus, immunotherapy

## Abstract

**Background:**

Trastuzumab-containing chemotherapy is the first-line treatment for advanced gastric cancer (GC) with HER2 positive. Although PD-1 inhibitors significantly improved the outcome of GC patient’s refractory to previous chemotherapy regimens, few studies explore the role of anti-PD-1 therapy overcomes resistance to trastuzumab plus chemotherapy in advanced Epstein-Barr Virus-associated gastric cancer (EBVaGC) with PD-L1 and HER2 positive.

**Case Presentation:**

We report a case of advanced EBVaGC in a 45-year-old man presenting with fatigue, dysphagia, and weight loss for several months. Initial endoscopy revealed a large tumor at the gastroesophageal junction. Computed tomography revealed GC accompanied by multiple lymph nodes and hepatic and pulmonary metastases. The immunohistochemistry indicated that HER-2 and PD-L1 were overexpressed, and tumor cells were positive for EBV-encoded small RNA (EBER) by *in situ* hybridization. Trastuzumab plus DCS was started as first-line chemotherapy with a PFS of 4 months and shifted to trastuzumab plus FOLFIRI or gemcitabine as second-/third-line therapy. After five-cycle nivolumab monotherapy, the patient received partial response and was treated with total radical gastrectomy plus sequential radiotherapy. He continued the postoperative immunotherapy over 30 cycles with a PFS of 28 months. Due to a new abdominal lymph node metastasis confirmed by PET-CT, he received toripalimab as the next-line treatment and achieved complete remission as the best objective response.

**Summary:**

We presented an advanced HER2-positive EBVaGC patient with PD-L1 high expression, refractory to trastuzumab plus chemotherapy, and had a durable clinical benefit sequence with a single dose of the PD-1 inhibitor.

## Introduction

Gastric cancer (GC) remains a major public health problem in China, with an estimated 396,500 new cases and 288,500 deaths in 2016 (1). For advanced GC with human epidermal growth factor receptor 2 (HER2) positive, the combination of trastuzumab plus chemotherapy has become the first-line treatment option, based on the results of the Trastuzumab for Gastric Cancer (ToGA) study (2). In the intent-to-treat population of the ToGA study, the median overall survival is 13.8 months in the trastuzumab plus chemotherapy arm and 11.1 months in the chemotherapy-only arm. When patients with advanced GC are refractory to two or more previous chemotherapy regimens, nivolumab is recommended and significantly improves their poor prognosis (3).

Epstein–Barr virus-associated gastric cancer (EBVaGC) was one of four classifications of GC, with frequent mutations of PIK3CA, ARID1A, and BCOR, and PD-L1 and PD-L2 amplification (4); it accounts for up to 10% of its molecular subtypes (5). A previous study has demonstrated that EBVaGC has the best prognosis among the four molecular subtypes and the effectiveness of adjuvant chemotherapy. Due to the increased expression of PD-L1, immunotherapy has aroused the greatest clinical interest for EBVaGC. Recently, a phase II study showed a 100% response rate to pembrolizumab among patients with metastatic EBVaGC (6). In this study, 29(29/61, 47.5%)patients received pembrolizumab as a third-line treatment, and 6 (6/61, 9.8%) patients were EBV-positive. However, little literature explores the response to PD-1 inhibition for metastatic EBVaGC refractory to two or more previous regimens of trastuzumab-contained chemotherapy. In this study, we presented a HER2-positive advanced EBVaGC patient with PD-L1 high expression, refractory to trastuzumab plus chemotherapy, and had a significant clinical benefit sequence with a single dose of nivolumab.

## Case presentation

**Figure 1:**
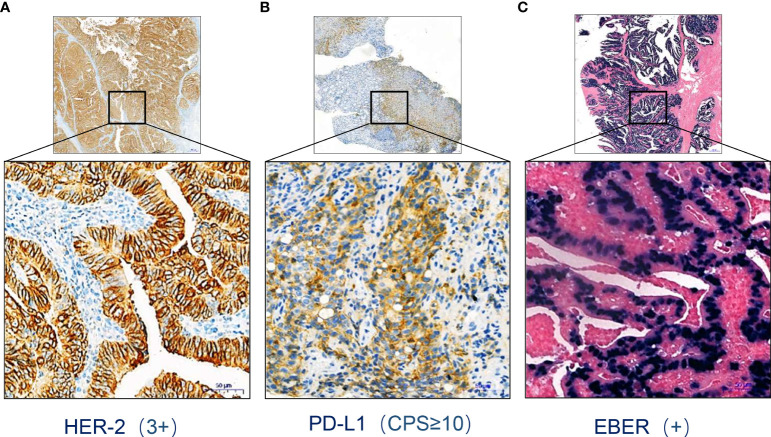
HER2 **(A)**, PD-L1 **(B)**, and EBER **(C)** staining of the primary tumor. Scale bars: 50 µm.

A 45-year-old man was diagnosed with advanced EBVaGC (TNM stage IV), presenting with fatigue, dysphagia, and weight loss for several months. Moreover, he underwent esophagogastroduodenoscopy (EGD) at a gastrointestinal clinic, where a large tumor at the gastroesophageal junction was detected, with the invasion of the lower esophageal sphincter, gastric body, and antrum. Pectoral and abdominal contrast-enhanced computerized tomography (CT) suggested uneven thickening and strengthening of the gastric body and cardial wall, possibly accompanied by multiple lymph nodes adjacent to the lesser gastric curvature and hepatic/pulmonary metastases. Subsequent pathological examination of the biopsy showed moderately to poorly differentiated adenocarcinoma. The immunohistochemistry indicated that HER-2 and PD-L1 were overexpressed (**Figures 1A, B**). Meanwhile, an EBV-encoded RNA (EBER) assay showed positive staining parallel to the tumor harboring EBV infection (**Figure 1C**).

The patient was treated with trastuzumab plus DCS (docetaxel + cisplatin+S-1) for six cycles as first-line treatment from September 7, 2016, to December 7, 2016, achieving a stable disease as the best objective response. During this period, the patient suffered from bone marrow suppression (CTCAE 4.1 Grade 3) and was treated with a recombinant human granulocyte colony-stimulating factor. On January 7, 2017 (PFS = 4 months), the lesion adjacent to lesser gastric curvature progression occurred (same known lesion of 64 mm, ECOG performance status 1 confirmed), and a new lesion of 11 mm on the left rectum. After that, the patient started the second-line treatment with trastuzumab plus FOLFIRI (folinic acid + fluorouracil + irinotecan) administered in a 28 days cycle. After 9 cycles, he obtained a stable disease and received microwave ablation of liver metastases on July 17, 2017. He received 11-cycle chemotherapy until September 8, 2017, due to progressive disease based on Response Evaluation Criteria in Solid Tumors (RECIST) 1.1. From Oct.11, 2017, to November 6, 2017, the patient was referred for trastuzumab plus gemcitabine with a 21-day cycle. Unfortunately, treatment was interrupted due to his poor clinical condition (poorly controlled cancer pain and ECOG performance status 4) and new liver metastatic tumors confirmed by pectoral and abdominal CT with contrast on December 9, 2017.

**Figure 2:**
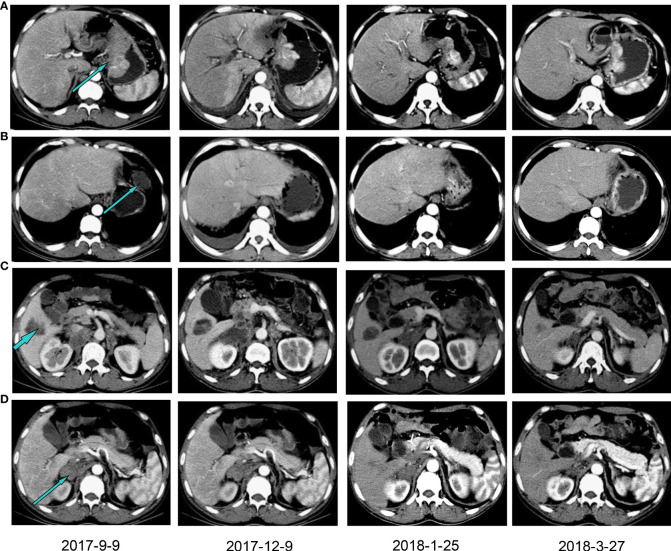
Computed tomography with contrast showing the patient’s lesions before and after PD-1 inhibitor treatment. The lesions in lesser gastric curvature **(A)**, left front of the gastric body **(B)**, right lobe of the liver **(C)** and right adrenal **(D)**. Patients were treated with a PD-1 inhibitor from December 2017.

**Figure 3:**
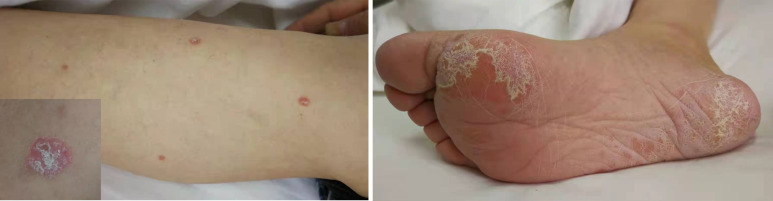
The management of nivolumab-related exfoliative dermatitis eruption.

Therefore, the patient received nivolumab (200mg, 14 days per cycle) monotherapy for five cycles as his four-line treatment. One cycle of immunotherapy later, his cancer pain was well controlled without nonsteroidal anti-inflammatory drugs. His performance status was significantly improved (ECOG PS 1), as well as his sleep quality and appetite. On January 25 and March 27, 2018, CT scans (with contrast) showed a partial response and a steadily shrinking of the known lesions, including the lesser gastric curvature (**Figure 2A**), left front of the gastric body (**Figure 2B**), right lobe of the liver (**Figure 2C**) and right adrenal (**Figure 2D**), but still in TNM stage IV. **Figure 2B** also showed that the new bilateral pleural effusion (2017–9–9) was completely absorbed after nivolumab treatment. Exfoliative dermatitis eruption was developed on his lower extremities and palmoplantar (**Figure 3**) after 40 days of PD-1 inhibitor treatment. However, two months later, the eruption completely disappeared, and no trace of symptoms was observed. According to the decisions from multidisciplinary teams, the patient received total radical gastrectomy for gastric carcinoma on July 25, 2018, plus sequential radiotherapy (60Gy/30F for visible abdomen metastasis). Postoperative pathological analysis revealed tumor regression grade 3 of Mandard (five, three-tier) system.

The patient continued the postoperative immunotherapy in the First Hospital of Yulin City. A regular serum tumor biomarkers and radiographic evaluation in our outpatient department confirmed no evidence of disease progression with more than 30-cycle nivolumab treatment until April 1, 2020. Due to a new abdominal lymph node metastasis confirmed by PET-CT, he received toripalimab as the next-line treatment and achieved complete remission as the best objective response.

## Tumor biomarker and genetic analyses

**Figure 4:**
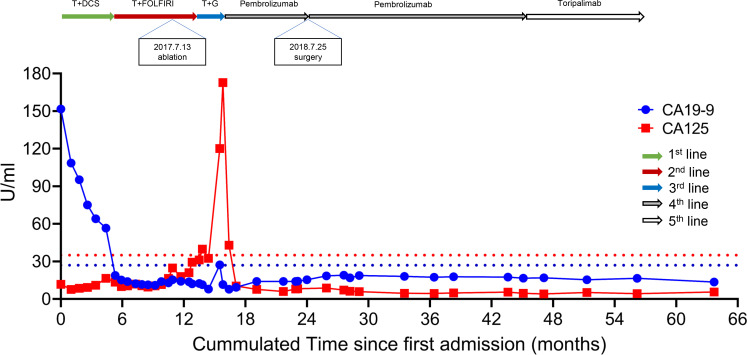
Dynamics of cancer antigen 19-9/125 levels during the entire disease course. T, Trastuzumab; DCS, docetaxel + cisplatin+S-1; FOLFIRI, folinic acid + fluorouracil + irinotecan.

**Table S1** showed the somatic variants detected in the 1000-gene next-generation sequencing panel. It verified four gene mutations, a low tumor mutation burden (TMB) with 7.76 Muts/Mb, and microsatellite stable (MSS). **Figure 4** displayed the trajectories of two tumor biomarkers during the whole period of therapy. For the blue, sharp-falling curve, CA19-9 declined rapidly from the elevated baseline level (151.7 U/mL) toward the normal range (< 27 U/mL) within six months of the first-line trastuzumab-containing treatment and then kept stable. For the red, later-rising curve, CA125 kept stable in the normal range from (< 35 U/mL) from pre-treatment to third-line therapy, increased rapidly to the highest level (172.8 U/mL) when abdominal CT confirmed disease progression with contrast on December 9, 2017, and sharply fell toward the normal range within 1 month of four-line treatment of PD-1 inhibitor.

## Discussion

This study presented a case report about a metastatic EBVaGC patient who significantly benefits from the 4th line of a single dose of nivolumab, which is refractory to trastuzumab across the first to third lines of chemotherapy beyond progression. In this case, patients received a stable disease as the best objective response during the trastuzumab-containing chemotherapy. Although his performance status was poor (PS score 4) and accompanied by malignant pleural effusion, his condition quickly improved with tumor shrinkage to PR and complete absorption of pleural effusion after three cycles of nivolumab monotherapy. To the best of our knowledge, this is the first report to show the long-term response of PD-1 inhibitors after resistance to trastuzumab-containing chemotherapy in advanced EBVaGC with PD-L1 and HER2 positive.

In clinical practice, trastuzumab-containing chemotherapy is the first-line treatment for the advanced GC with HER2 positive, and nivolumab for HER2 negative disease with PD-L1 CPS ≥5 (7). Furthermore, a phase 2 trial proved that pembrolizumab plus trastuzumab-containing chemotherapy was a safe and promising treatment in HER2-positive metastatic esophagogastric cancer (8). The results were further confirmed by the KEYNOTE-811 study (9), a randomized, double-blind, placebo-controlled phase III trial that significantly added pembrolizumab to trastuzumab and chemotherapy to improve the objective response rate in the unresectable HER2-positive metastatic esophagogastric cancer. In the second-line and subsequent therapy, pembrolizumab is recommended for MSI-H/dMMR or TMB-H (>10 mutations/Mb) tumors (7). However, the patient who significantly benefited from the PD-1 inhibitor had PD-L1 CPS =10, HER2 3+, EBER + but TMB-L (7.76 Muts/Mb). Besides, the PFS of our case (beyond 20 months) was more prominent, and it could be translated into long-term survival. Similar results had been reported (10), which may attribute to the unique characteristics of EBV-related cancers. The present case indicated EBV was a strong biomarker for the immunotherapy of GC. It was consistent with the results from a phase II study with a 100% response rate to pembrolizumab for metastatic EBVaGC (6). Dynamics of CA199 and CA125 levels were shown during the entire disease course, and the peak shape of the CA125 trajectory indicated a quick response to PD-1 inhibitor with complete absorption of pleural effusion. A similar trajectory of CA199 was observed in a HER2-positive, metastatic gallbladder cancer patient’s complete response to pembrolizumab after resistance to trastuzumab plus chemotherapy (11). CA199/CA125 serological response (12) could be associated with durable benefits from pembrolizumab.

In summary, we showed that an advanced EBVaGC patient received a long response to nivolumab monotherapy after failure of trastuzumab-containing chemotherapy with PD-L1 and HER2 positive. His condition and performance status quickly improved after three cycles of nivolumab treatment. Our case demonstrated that PD-1 antibody could produce robust and durable responses in metastatic HER2-positive EBVaGC resistant to trastuzumab-containing chemotherapy. In the future, PD-1 inhibitor combination of trastuzumab is warranted as the frontline treatment for advanced GC patients with PD-L1 and HER2 positive, especially for EBVaGC.

## Data availability statement

The original contributions presented in the study are included in the article/**Supplementary Material**. Further inquiries can be directed to the corresponding author.

## Ethics statement

The patient agreed and submitted a written informed consent to allow publication of the details of his case. The Ethics Committee approved this case report of Xijing Hospital.

## Author contributions

All authors contributed to the article and approved the submitted version.

## Funding

This work was supported by the National Major Research and the Innovation Program of China (Grant No. 2016YFC1303200), the National Key R&D Program of China (Grant No. 2017YFC0908300), and the National Natural Science Foundation of China (Grant No. 81972761)

## Conflict of interest

The authors declare that the research was conducted in the absence of any commercial or financial relationships that could be construed as a potential conflict of interest.

## Publisher’s note

All claims expressed in this article are solely those of the authors and do not necessarily represent those of their affiliated organizations, or those of the publisher, the editors and the reviewers. Any product that may be evaluated in this article, or claim that may be made by its manufacturer, is not guaranteed or endorsed by the publisher.
