# Supplementary material for: Case report: Long response to PD-1 blockade after failure of trastuzumab plus chemotherapy in advanced Epstein-Barr virus-associated gastric cancer
Source: Front Immunol. 2022 Oct 24;13:1003859. doi: 10.3389/fimmu.2022.1003859 (PMC9639782; doi:10.3389/fimmu.2022.1003859)
Supplement: Supplementary file 1 [file DataSheet_1.docx]

**Table S1. Somatic Variants Detected in the 1000-Gene Next-Generation Sequencing Panel**

| **Gene** | Reference Sequence | Variant | Amino Acid | Variant Allele Frequency (%) |
| --- | --- | --- | --- | --- |
| **KRAS** | NM_033360 | c.35C>T | p.G12D | **7.80%** |
| **TP53** | NM_000546 | c.638C>T | p.R213Q | **20.90%** |
| **CTNNB1** | NM_001904 | c.121A>G | p.T41A | **8.70%** |
| **CTNNB1** | NM_001904 | c.94G>T | p.D32Y | **15.50%** |
